# Complex associations of adverse childhood and adulthood experiences with incident depressive symptoms in middle-aged and older Chinese adults

**DOI:** 10.1186/s40359-026-04333-8

**Published:** 2026-03-14

**Authors:** Wentao Huang, Jianfei Li, Ruijie Wang, Jinling Zhang, Xin Ye, Mianmian Chen

**Affiliations:** 1 Department of Geriatric Medicine, Shantou Central Hospital, No. 114 Waima Road, Shantou, 515041 China; 2 Department of Obstetrics, Shantou Central Hospital, No. 114 Waima Road, Shantou, 515041 China

**Keywords:** Adverse childhood experiences, Adverse adulthood experiences, Incident depressive symptoms, Middle-aged and older Chinese adults

## Abstract

**Backgrounds:**

The associations of adverse childhood experiences (ACEs) and adverse adulthood experiences (AAEs) with incident depressive symptoms remain incompletely understood. The study sought to explore the complex associations of ACEs and AAEs with incident depressive symptoms among middle-aged and older adults.

**Methods:**

Data of 4516 participants aged ≥ 45 years were sourced from the China Health and Retirement Longitudinal Study. Information on ACEs, AAEs, depressive symptoms, sociodemographic profiles, health behaviors, self-reported chronic diseases, retirement status, and adulthood social support was collected. Modified Poisson regression analysis, Karlson-Holm-Breen mediation analysis and interaction analysis were conducted.

**Results:**

The overall numbers of ACEs (RR: 1.12; 95% CI: 1.08, 1.16) and AAEs (RR: 1.10; 95% CI: 1.06, 1.14) were independently associated with an increased risk of incident depressive symptoms. Significant dose-response relationships were detected between the numbers of ACEs (P-trend < 0.001) and AAEs (P-trend < 0.001) and the risk of incident depressive symptoms. The overall AAEs mediated 5.78% of the total effect of ACEs on incident depressive symptoms. No significant multiplicative (RR: 0.99; 95% CI: 0.77, 1.25) or additive [RERI (95% CI): 0.02 (-0.27, 0.30); AP (95% CI): 0.01 (-0.19, 0.21); SI (95% CI): 1.04 (0.49, 2.20)] interactions were detected between ACEs and AAEs on incident depressive symptoms.

**Conclusions:**

Both ACEs and AAEs were independently associated with an increased risk of incident depressive symptoms in a dose-response manner, with AAEs partially mediating the total effect of ACEs on incident depressive symptoms. These findings support preventive strategies targeting ACEs and AAEs to mitigate the risk of incident depressive symptoms in later adulthood.

**Supplementary Information:**

The online version contains supplementary material available at 10.1186/s40359-026-04333-8.

## Introduction

Depression is a highly prevalent affective disorder resulting from the complex interaction of genetic, biological, and psychosocial factors [1]. In accordance with the Global Burden of Disease Study 2021, there were an estimated 332 million cases of depressive disorders worldwide in that year [2]. This affective disorder also poses a substantial public health challenge in China, with an epidemiological study reporting a 6.8% lifetime prevalence among Chinese adults [3]. Depression is established as an independent risk element for numerous chronic conditions, encompassing autoimmune diseases, cardiovascular diseases and other systemic diseases [4, 5]. It constitutes the second highest contributor to global disability burden, driving elevated healthcare utilization and costs [2, 6]. Given the substantial medical, economic, and social burdens imposed by depression, it is imperative to pinpoint intervenable determinants and develop targeted prevention strategies.

Beck’s cognitive model of depression postulates that depression stem from individuals’ negative interpretations of experiences. These distorted cognitive patterns are rooted in maladaptive worldviews, which may develop through exposure to adverse experiences across the life course, particularly adverse childhood experiences (ACEs) and adverse adulthood experiences (AAEs) [7]. ACEs encompass diverse stressful and traumatic events during childhood and adolescence, spanning neglect, abuse, household dysfunction, and other forms of psychosocial adversity [8, 9]. Recent epidemiological studies have increasingly established ACEs as an independent etiological contributor to depression [10–13]. The hypothesized mechanisms span both neurobiological and psychosocial pathways [14, 15]. Similarly, AAEs, referring to a broad range of stressful and traumatic events occurring during adulthood, include adversities such as severe health crises, discrimination, traumatic bereavement, and socioeconomic instability [16, 17]. Multiple studies have found that AAEs were also a key etiological contributor to depression [17–21].

However, critical knowledge gaps persist in the current literature. First, although extant evidence indicates that exposure to ACEs may heighten the probability of encountering AAEs [17, 18], and that AAEs may mediate the effect of ACEs on health outcomes [22], the potential mediation by AAEs linking ACEs to depression remains understudied. Second, prior studies have yielded divergent empirical support on how ACEs and AAEs interact to shape depression risk. Some studies have validated the stress sensitization hypothesis, proposing that ACEs may amplify susceptibility to depression when individuals face subsequent AAEs [19, 23]. In contrast, other studies have substantiated the stress inoculation framework, arguing that ACEs may enhance stress-adaptive capacities, thereby protecting against depression triggered by AAEs [20, 24]. Third, middle-aged and older adults bear a significant burden of depression [25], which in this population is associated with elevated risks of physical disability, cognitive decline, and premature mortality [26–28]. Against the backdrop of accelerating global population aging, identifying modifiable risk factors, such as adversities occurring across the life course, has become an urgent public health priority. Nonetheless, research specifically examining the combined influence of ACEs and AAEs on depression in this population remains limited [19–21]. These knowledge gaps highlight the need for more systematic studies to address outstanding questions in this field.

The present study sought to achieve three primary objectives: first, to assess the associations of ACEs and AAEs with incident depressive symptoms; second, to examine whether AAEs mediated the association between ACEs and incident depressive symptoms; third, to explore the potential interaction between ACEs and AAEs on incident depressive symptoms.

## Methods

### Data source

The data were sourced from the China Health and Retirement Longitudinal Study (CHARLS), a longitudinal national study tracking multidimensional trajectories of health, socioeconomic status, and social engagement among Chinese adults aged ≥ 45 years [29]. The detailed study design has been documented in prior publications [9, 29, 30]. Briefly, 17,708 participants across 450 villages or communities in 28 Chinese provinces were recruited in the baseline survey in 2011. The participants underwent follow-ups biennially to triennially, with supplemental recruitment conducted during each survey wave. Up to present, four survey waves have been conducted in 2013, 2015, 2018, and 2020, respectively. Moreover, the life history survey was conducted in 2014, which retrospectively documented lifetime trajectories for surviving participants from the 2011 and 2013 surveys. CHARLS received ethical clearance from Peking University’s Institutional Review Board. All individuals submitted written informed consent preceding study involvement. The present study incorporated data from the 2015 and 2020 surveys with the 2014 life history survey. As a secondary analysis of repository-sourced data, this study received exemption from additional ethics review and consent requirements under the governing ethics framework of the London School of Economics and Political Science [31].

### Study population

**Fig. 1:**
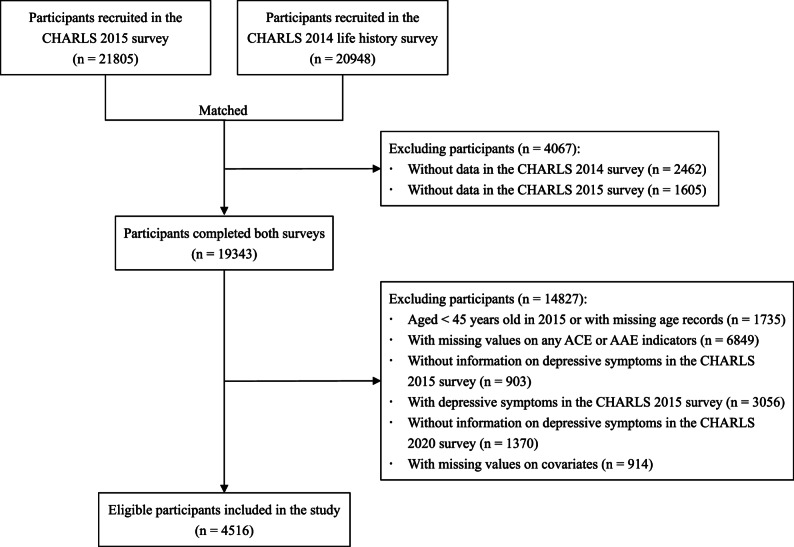
Flow diagram for the selection of the study participants. CHARLS, China Health and Retirement Longitudinal Study; ACE, adverse childhood experience; AAE, adverse adulthood experience

Figure 1 illustrates the detailed process of study population selection. Overall, 21,805 and 20,948 participants were recruited in the 2015 survey and the 2014 life history survey, respectively. After performing 1:1 matching of dual-survey completers, 19,343 participants were initially included. Subsequently, we excluded those aged < 45 years old in 2015 or with missing age records (*n* = 1735), those with missing values on any ACE or AAE indicators (*n* = 6849), those without information on depressive symptoms in the 2015 survey (*n* = 903), those with depressive symptoms in the 2015 survey (*n* = 3056), those without information on depressive symptoms in the 2020 survey (*n* = 1370), and those with missing values on any covariates (*n* = 914). Ultimately, 4516 participants were retained for the final analysis.

### ACEs and AAEs

Consistent with prior studies, 12 ACEs were extracted from the 2014 life history survey: physical abuse, emotional neglect, domestic violence, incarcerated household member, household substance use, household mental illness, parental separation or divorce, parental disability, parental death, death of siblings, unsafe neighborhood and bullying [9, 30, 32]. The specific definitions and questionnaire items for each ACE indicator are detailed in Supplementary Table 1. Individuals’ responses to each item underwent dichotomous coding and then aggregated into a cumulative ACE score (range: 0–12). Using this index, participants were further classified into 5 groups: 0, 1, 2, 3, and ≥ 4. Simultaneously, 7 AAEs were extracted from the 2014 life history survey building upon prior studies: having experienced the death of a child, having experienced lifetime discrimination, ever being confined to bed or home for ≥ 1 month, ever being hospitalized for ≥ 1 month, ever being hospitalized ≥ 3 times, ever leaving a job due to health conditions, and physical injury [16, 22]. Supplementary Table 1 provides the detailed definitions and corresponding questionnaire items for each AAE indicator. Responses to each item were dichotomized and aggregated into a cumulative AAE score (range: 0–7) for each participant. Participants were further classified into 5 group: 0, 1, 2, 3, and ≥ 4.

### Depressive symptoms

Depressive symptoms were evaluated using the 10-item Center for Epidemiologic Studies Depression Scale [33], a psychometrically robust instrument validated in Chinese adult populations [34, 35]. The scale comprises 10 questions regarding participants’ feelings and behaviors during the preceding week, with 8 negatively worded items and 2 positively worded items. Responses to each item were scored on a 4-point Likert scale: 0 (< 1 day), 1 (1–2 days), 2 (3–4 days), and 3 (5–7 days). The two positively worded items underwent inverse coding prior to summation. A composite score (range: 0–30) was derived by aggregating all items scores. Scores meeting or exceeding the clinical threshold of 10 signified the presence of depressive symptoms [33–35]. Incident depressive symptoms were defined as the absence of depressive symptoms at the 2015 baseline survey and the presence of depressive symptoms at the 2020 follow-up survey. Since depression history information was not available in the CHARLS data, it was not considered in the definition of incident depressive symptoms in this study.

### Covariates

In the 2015 survey, sociodemographic profiles, health behaviors, and self-reported chronic diseases were ascertained through structured in-person interviews. The sociodemographic profiles covered age, sex, residential area, marital status, and education level. The health behavior included smoking and drinking status, and social activity participation. Self-reported chronic diseases encompassed diabetes, hypertension, dyslipidemia, heart problems, stroke, kidney diseases, lung diseases, digestive diseases, liver diseases, cancer, rheumatism, asthma, psychiatric problems, and memory-related diseases. Participants presenting with any of the aforementioned chronic diseases were defined as having chronic diseases. Additionally, retirement status and adulthood social support, including economic, non-economic, and emotional aspects, were collected in this study. Responses to the three questions concerning social support underwent dichotomous coding and aggregated into a composite score (range: 0–3).

### Statistical analysis

First, cohort baseline characteristics were stratified by incident depressive symptoms status, ACEs or AAEs exposure levels, with between-group variations assessed by appropriate statistical methods. The characteristics were also compared between included and excluded participants. The distributional normality of continuous variables was evaluated through Q-Q plots. Normally distributed variables were expressed as mean ± standard deviation with one-way ANOVA for groupwise comparisons; non-normally distributed variables were expressed as median (interquartile range) with Kruskal-Wallis test for groupwise comparisons. Categorical variables were presented as frequencies (percentages) and assessed by chi-square test for intergroup differences. To assess trends in characteristics across different ACEs and AAEs groups, polynomial contrasts were used for continuous variables and Mantel-Haenszel text was used for categorical variables. Moreover, a linear regression analysis with age and sex adjustment was conducted to evaluate the association between the overall number of ACEs and that of AAEs.

Second, the incidence rates of depressive symptoms were determined for each ACEs and AAEs group. Modified Poisson regression models with robust standard errors were constructed to directly estimate relative risks (RRs) with 95% confidence intervals (95% CIs) for the associations of ACEs and AAEs with incident depressive symptoms. This approach was chosen because the outcome was not rare in our cohort, and RRs provide a more interpretable measure of association for public health implications than odds ratios derived from logistic regression. In the crude model, no confounding variables were included for adjustment. Model 1 adjusted for age and sex. Model 2 further adjusted for residential area, marital status, education level, smoking status, drinking status, social activity participation, presence of chronic diseases, retirement status, and adulthood social support. Trend tests were subsequently conducted to evaluate potential dose-response relationships.

Third, the Karlson-Holm-Breen (KHB) method was employed to examine whether overall AAEs mediated the association between ACEs and incident depressive symptoms [36]. The KHB method, widely recognized for its ability to address rescaling bias in nonlinear models, disaggregates total effects into direct and indirect effect pathways by residualizing intermediate variables [36]. In this analysis, all effects were adjusted for every covariate included in model 2. Additionally, a supplementary mediation analysis was performed to specifically assess the mediating role of AAEs in the association between high-dose ACEs and depressive symptoms. In this analysis, ACEs were specified as a binary variable comparing individuals with high exposure (≥ 4 ACEs) to those with no exposure (0 ACEs), using the same KHB method and adjustment strategy.

Fourth, we explored the potential interaction between ACEs and AAEs through dichotomizing exposures (0: no ACE or AAE indicator; ≥ 1: at least one ACE or AAE indicator) and then partitioning participants into four mutually exclusive groups based upon combinatorial exposure status. For the assessment of multiplicative interaction, the product term (ACEs × AAEs) was incorporated into model 2, and its RR with 95% CI was used to quantify the effect. Additive interaction was evaluated by the relative excess risk due to interaction (RERI) with 95% CI, the attributable proportion (AP) with 95% CI, and the synergy index (SI) with 95% CI. These metrics were calculated using the regression coefficients and covariance matrix obtained from model 2 [37].

Fifth, subgroup analyses were performed to explore heterogeneity across age categories [< 60 years (middle-aged adults) and ≥ 60 years (older adults)] and sex classifications (males and females). To test for interaction, we introduced multiplicative interaction terms (e.g., age group × ACEs, sex × ACEs) into the main regression models. The statistical significance of the interaction was evaluated using the Wald test.

Analyses were conducted with STATA 18.0 and SPSS 26.0. Statistical significance was defined as two-sided *P* < 0.05.

## Results

Supplementary Table 2 shows the baseline characteristics of included and excluded participants. Statistically significant differences were found for all characteristics except social support. Table 1 summarizes the baseline characteristics of the study population grouped according to the occurrence of depressive symptoms. Among 4516 analyzed participants, 1162 (25.7%) developed depressive symptoms during the follow-up period. Compared with those without depressive symptoms, participants with incident depressive symptoms exhibited distinct characteristics: they were older, more likely to be female, and had higher ACEs/AAEs scores, higher chronic disease burden, lower education attainment, lower rates of urban residence, marriage, smoking, drinking and social activity participation, a lower likelihood of being retired, and less social support. The baseline characteristics of the study population grouped by ACEs and AAEs exposure levels are presented in Supplementary Tables 3–4, respectively. 73.2% (*n* = 3304) of participants reported ≥ 1 ACE indicator and 33.0% (*n* = 1489) reported ≥ 1 AAE indicator. The overall number of ACEs showed a significant positive relationship with that of AAEs (β: 0.094; 95% CI: 0.069, 0.120).

**Table 1:** Baseline characteristics of the study population by depressive symptoms status

Characteristics^a^	Overall	Depressive symptoms	No depressive symptoms	*P* for difference
	(*n* = 4516)	(*n* = 1162)	(*n* = 3354)	
Age, mean ± SD, years	58.37 ± 7.97	58.80 ± 8.20	58.22 ± 7.88	0.036
Sex, n (%)				< 0.001
Male	2440 (54.0)	482 (41.5)	1958 (58.4)	
Female	2076 (46.0)	680 (58.5)	1396 (41.6)	
Residential area, n (%)				< 0.001
Urban	1808 (40.0)	354 (30.5)	1454 (43.4)	
Rural	2708 (60.0)	808 (69.5)	1900 (56.7)	
Marital status, n (%)				0.016
Married	3990 (88.4)	1004 (86.4)	2986 (89.0)	
Other marital status	526 (11.6)	158 (13.6)	368 (11.0)	
Educational level, n (%)				< 0.001
No formal education	687 (15.2)	271 (23.3)	416 (12.4)	
Primary school or below	1724 (38.2)	508 (43.7)	1216 (36.3)	
Middle school	1316 (29.1)	259 (22.3)	1057 (31.5)	
High school or above	789 (14.5)	124 (10.7)	665 (19.8)	
Smoking status, n (%)				< 0.001
Nonsmoker	2374 (52.6)	702 (60.4)	1672 (49.9)	
Former smoker	768 (17.0)	161 (13.9)	607 (18.1)	
Current smoker	1374 (30.4)	299 (25.7)	1075 (32.1)	
Drinking status, n (%)				< 0.001
Never drinker	2250 (49.8)	671 (57.8)	1579 (47.1)	
Former drinker	448 (9.9)	117 (10.1)	331 (9.9)	
Current drinker	1818 (40.3)	374 (32.2)	1444 (43.1)	
Social activity, n (%)				< 0.001
No	2095 (46.4)	606 (52.2)	1489 (44.4)	
Yes	2421 (53.6)	556 (47.9)	1865 (55.6)	
Social support, median (IQR)	0 (1.00)	0 (0)	0 (1.00)	< 0.001
Chronic diseases, n (%)				< 0.001
No	1082 (24.0)	223 (19.2)	859 (25.6)	
Yes	3434 (76.0)	939 (80.8)	2495 (74.4)	
Retire, n (%)				< 0.001
No	3822 (84.6)	1039 (89.4)	2783 (83.0)	
Yes	694 (15.4)	123 (10.6)	571 (17.0)	
ACEs, median (IQR)	1.00 (2.00)	1.00 (1.00)	1.00 (2.00)	< 0.001
ACEs group, n (%)				< 0.001
0	1212 (26.8)	274 (23.6)	938 (28.0)	
1	1478 (32.7)	340 (29.3)	1138 (33.9)	
2	998 (22.1)	276 (23.8)	722 (21.5)	
3	514 (11.4)	146 (12.6)	368 (11.0)	
≥ 4	314 (7.0)	126 (10.8)	188 (5.6)	
AAEs, median (IQR)	0 (1.00)	0 (1.00)	0 (1.00)	< 0.001
AAEs group, n (%)				< 0.001
0	3027 (67.0)	720 (62.0)	2307 (68.8)	
1	797 (17.7)	226 (19.5)	571 (17.0)	
2	327 (7.2)	94 (8.1)	233 (7.0)	
3	211 (4.7)	64 (5.5)	147 (4.4)	
≥ 4	154 (3.4)	58 (5.0)	96 (2.9)	

*Abbreviations*: *ACEs* Adverse childhood experiences, *AAEs* Adverse adulthood experiences, *SD* Standard deviation, *IQR* Interquartile range

^a^Normally distributed variables are presented as mean ± SD, non-normally distributed variables are presented as median (IQR), and categorical variables are presented as n (%)

As depicted in Table 2, the incidence rates of depressive symptoms escalated monotonically with the numbers of ACEs and AAEs. After comprehensive covariate adjustment in model 2, the overall numbers of ACEs (RR: 1.12; 95% CI: 1.08, 1.16) and AAEs (RR: 1.10; 95% CI: 1.06, 1.14) were independently associated with an increased risk of incident depressive symptoms. Relative to participants unexposed to ACEs, the risk of incident depressive symptoms showed a graded increase from 1.22 (95% CI: 1.06, 1.40) for those with two ACEs to 1.71 (95% CI: 1.44, 2.02) for those with ≥ 4 ACEs. When compared with participants unexposed to AAEs, the risk of incident depressive symptoms reached 1.24 (95% CI: 1.01, 1.51) for those with three AAEs and 1.57 (95% CI: 1.28, 1.93) for those with ≥ 4 AAEs. Significant dose-response relationships were detected for the numbers of ACEs (P-trend < 0.001) and AAEs (P-trend < 0.001) with the risk of incident depressive symptoms.

**Table 2:** Incidence of depressive symptoms and associations of ACEs and AAEs with incident depressive symptoms

	Incidence rates of depressive symptoms, %	RR (95% CI)		
		Crude model	Adjusted model 1^a^	Adjusted model 2^b^
ACEs indicators^c^ (1-unit per increasing)	NA	1.13 (1.09, 1.17)	1.14 (1.10, 1.18)	1.12 (1.08, 1.16)
No. of ACEs indicators				
0	22.6	1.00 (reference)	1.00 (reference)	1.00 (reference)
1	23.0	1.02 (0.88, 1.17)	1.05 (0.91, 1.20)	1.04 (0.91, 1.19)
2	27.7	1.22 (1.06, 1.41)	1.26 (1.09, 1.46)	1.22 (1.06, 1.40)
3	28.4	1.26 (1.06, 1.49)	1.35 (1.14, 1.60)	1.32 (1.12, 1.56)
≥ 4	40.1	1.77 (1.50, 2.11)	1.82 (1.54, 2.16)	1.71 (1.44, 2.02)
P for trend	< 0.001	< 0.001	< 0.001	< 0.001
AAEs indicators^c^ (1-unit per increasing)	NA	1.11 (1.07, 1.15)	1.13 (1.09, 1.18)	1.10 (1.06, 1.14)
No. of AAEs indicators				
0	23.8	1.00 (reference)	1.00 (reference)	1.00 (reference)
1	28.4	1.19 (1.05, 1.35)	1.21 (1.06, 1.37)	1.13 (0.99, 1.28)
2	28.7	1.21 (1.01, 1.45)	1.24 (1.03, 1.49)	1.15 (0.96, 1.38)
3	30.3	1.28 (1.03, 1.58)	1.32 (1.07, 1.64)	1.24 (1.01, 1.53)
≥ 4	37.7	1.58 (1.28, 1.96)	1.76 (1.43, 2.17)	1.57 (1.28, 1.93)
P for trend	< 0.001	< 0.001	< 0.001	< 0.001

*Abbreviations*: *ACEs* Adverse childhood experiences, *AAEs* Adverse adulthood experiences, *RR* Relative risk, *95% CI* 95% confidence interval, *NA* Not available or not applicable

^a^Model 1 was adjusted for age and sex

^b^Model 2 was adjusted for age, sex, residential area, marital status, education level, smoking status, drinking status, social activity participation, presence of chronic diseases, retirement status, and adulthood social support

^c^Continuous variable

Table 3 presents significant mediation effects by overall AAEs in the association between ACEs and incident depressive symptoms. For each additional ACEs exposure, the mediation pathway through AAEs demonstrated a RR of 1.01 (95% CI: 1.002, 1.02), representing 5.78% of the total effect. For ≥ 1 ACEs exposure, the indirect effect RR was 1.02 (95% CI: 1.005, 1.03), with 8.58% mediation proportion. Additionally, a supplementary analysis comparing individuals with high-dose ACEs (≥ 4) to those with no ACEs revealed an indirect effect RR of 1.03 (95% CI: 1.003, 1.05), corresponding to a mediation proportion of 7.02%. The detailed results of this analysis are provided in the Supplementary Table 5.

**Table 3:** Mediating role of AAEs in the association between ACEs and incident depressive symptoms

	RR (95% CI)^a^			Mediation proportion, %
	Total effect	Direct effect	Indirect effect	
ACEs indicators^b^ (1-unit per increasing)	1.12 (1.07, 1.17)	1.11 (1.07, 1.16)	1.01 (1.002, 1.02)	5.78
No. of ACEs indicators				
0	1.00 (reference)	1.00 (reference)	1.00 (reference)	
≥ 1	1.19 (1.04, 1.37)	1.18 (1.03, 1.35)	1.02 (1.005, 1.03)	8.58

*Abbreviations*:*AAEs* Adverse adulthood experiences, *ACEs* Adverse childhood experiences, *RR* Relative risk, *95% CI* 95% confidence interval

^a^Models were adjusted for age, sex, residential area, marital status, education level, smoking status, drinking status, social activity participation, presence of chronic diseases, retirement status, and adulthood social support

^b^Continuous variable

Table 4 summarizes interaction analysis outcomes. For the multiplicative interaction, the RR of the product term in model 2 was 0.99 (95% CI: 0.77, 1.25). Concerning the additive interaction, the RERI, the AP, and the SI were 0.02 (95% CI: -0.27, 0.30), 0.01 (95% CI: -0.19, 0.21), and 1.04 (95% CI: 0.49, 2.20), respectively. These results demonstrated no statistically significant multiplicative or additive interactions existed between ACEs and AAEs on incident depressive symptoms.

**Table 4:** Interaction analysis of ACEs and AAEs on incident depressive symptoms

Interaction	RR (95% CI)^a^
Multiplicative interaction	
Product term (ACEs × AAEs)	0.99 (0.77, 1.25)
Additive interaction	
RERI	0.02 (-0.27, 0.30)
AP	0.01 (-0.19, 0.21)
SI	1.04 (0.49, 2.20)

*Abbreviations*:*ACEs* Adverse childhood experiences, *AAEs* Adverse adulthood experiences, *RR* Relative risk, *95% CI* 95% confidence interval, *RERI* Relative excess risk due to interaction, *AP* Attributable proportion, *SI* Synergy index

^a^Models were adjusted for age, sex, residential area, marital status, education level, smoking status, drinking status, social activity participation, presence of chronic diseases, retirement status, and adulthood social support

Subgroup analysis outcomes are detailed in Supplementary Tables 6–8. After covariate adjustment, the positive associations of both ACEs and AAEs with incident depressive symptoms were consistently observed across all subgroups. Specifically, the risk of depressive symptoms increased with each additional ACEs or AAEs indicator among both middle-aged (ACEs: RR: 1.14; 95% CI: 1.09, 1.19; AAEs: RR: 1.10; 95% CI: 1.04, 1.16) and older (ACEs: RR: 1.09; 95% CI: 1.03, 1.15; AAEs: RR: 1.10; 95% CI: 1.04, 1.16) adults, as well as among both males (ACEs: RR: 1.16; 95% CI: 1.09, 1.22; AAEs: RR: 1.12; 95% CI: 1.05, 1.19) and females (ACEs: RR: 1.10; 95% CI: 1.05, 1.15; AAEs: RR: 1.08; 95% CI: 1.03, 1.14). Tests for interaction indicated that neither age nor sex significantly modified the associations of ACEs or AAEs with incident depressive symptoms (Supplementary Table 6). Analyses exploring potential heterogeneity in mediation effects across subgroups revealed generally consistent but modest indirect pathways. The proportion of the total effect of ACEs (each additional exposure) mediated by AAEs was numerically higher among older adults (7.66%) and males (4.85%) compared to middle-aged adults and females, in whom the point estimates for the indirect effect were not statistically significant. When examining the association with exposure to any ACEs (≥ 1), the mediated proportions were not statistically significant in any subgroup (Supplementary Table 7). Aligning with the primary results, neither multiplicative nor additive interactions between ACEs and AAEs on incident depressive symptoms reached statistical significance in analyses stratified by age or sex (Supplementary Table 8).

## Discussion

This study demonstrated that both ACEs and AAEs were independently associated with an increased risk of incident depressive symptoms in middle-aged and older Chinese adults, exhibiting dose-response relationships with increasing exposure levels. AAEs partially mediated the association between ACEs and incident depressive symptoms. No significant multiplicative or additive interactions were detected between ACEs and AAEs on incident depressive symptoms.

Our study found that 73.2% of participants had experienced ACEs. The proportion is relatively higher compared to findings from studies in other countries [17, 21, 38, 39], reflecting a greater burden of ACEs within Chinese population. This phenomenon could be attributed to the nation’s unique historical contexts (e.g., widespread famine and sociopolitical upheavals in the mid-20th century) and cultural backgrounds (e.g., authoritative parenting styles emphasizing obedience). In contrast, only 33.0% of participants reported exposure to AAEs, a lower rate than that reported in most international studies [17, 21, 39]. This observation may, to some extent, reflect the progress and development of Chinese society. However, cross-study comparisons warrant cautious interpretation given methodological variations and sample heterogeneity across epidemiological investigations. In addition, the documented 25.7% depressive symptoms prevalence in this study aligns with findings from the China Family Panel Studies [40], reflecting to some extent the significant psychosocial burdens experienced by middle-aged and older adults during China’s societal transformation. Notably, the depressive symptom prevalence rates reported in these two studies is considerably higher than the depressive disorder prevalence reported by Lu J et al. [3], likely due to the latter’s use of DSM-IV criteria for diagnosing depressive disorders, rather than relying solely on CESD-10 to assess symptoms.

Existing literature has consistently identified ACEs and AAEs as independent risk elements for depression across diverse populations [17, 18, 20, 21, 39]. However, scarce research has specifically assessed these associations within middle-aged and older adults. Analysis of U.S. Health and Retirement Study demonstrated significant linkages between childhood adversity, adulthood adversity, and depression among older adults [21]. Similarly, a Japanese study in community-dwelling older adults found independent associations of childhood adversities and late-life stressors with the onset of depressive symptoms [20]. Our study extends this evidence by revealing ACEs and AAEs as independent risk elements for incident depressive symptoms among middle-aged and older Chinese adults, with a clear dose-response relationship. Although the potential utility of interventions targeting ACEs or AAEs for reducing the incidence of depression remains untested in randomized clinical trials, the remediable nature of ACE or AAE sequelae in later-life has emerged as a pivotal focus [41]. Our findings may facilitate the early identification of susceptible populations and guide the prioritization of preventive interventions for individuals exposed to ACEs or AAEs to safeguard mental health.

The precise mechanisms linking ACEs and AAEs to depression remain incompletely understood. Pathogenic mechanisms may involve hypothalamic-pituitary-adrenal axis dysregulation, chronic immuno-inflammatory activation, persistent oxidative stress, and neurostructural and functional alterations [14, 42, 43]. Furthermore, ACEs and AAEs correlate with compromised physical health, unhealthy behaviors, and poor interpersonal skills, which collectively establish predisposing pathways to depression [9, 15, 44].​ Thus, the above associations are biologically plausible, though empirical validation of underlying mechanisms is warranted.

Few investigations have established pathway models connecting ACEs, AAEs, and later depressive symptoms. A U.S. study among low-income women indicated that the ACEs-depression connection was mediated by adulthood adversity [17]. A Mexican adult cohort study ​demonstrated adulthood adversity mediating 47.5% of childhood adversity’s total effect on depression [39]. Consistent with the mentioned findings, the present study revealed that AAEs partially mediated the association between ACEs and incident depressive symptoms. The stress generation model potentially explains AAEs’ mediation effect. In particular, early-life adversity elevates vulnerability to subsequent adult stressors, with this cascade mediating ACEs-depression associations [21, 45, 46]. The significant positive relationship between the overall numbers of ACEs and AAEs detected in our study supports this conceptual model. Notably, the observed mediating effect of AAEs was weak, even in the high-dose ACEs (≥ 4) group. This limited mediation may be attributed to several factors. First, the specific adversities measured in the ACEs and AAEs scales likely represented distinct constructs, resulting in a relatively low correlation between them and thus a constrained indirect pathway. Second, the weak mediation suggests that the detrimental impact of ACEs may operate primarily through direct pathways, rather than being substantially channeled via the accumulation of AAEs. Finally, other complex mediating mechanisms might play more prominent roles in linking ACEs to later depressive symptoms [9, 47]. In-depth research on this topic is needed.

Furthermore, this study systematically evaluated both multiplicative and additive interactions between ACEs and AAEs on incident depressive symptoms, thereby addressing a critical methodological limitation in prior research that often focused exclusively on multiplicative interaction alone [20, 24]. Additive interaction assessment provides more clinically relevant insights into the biological synergism underlying disease pathogenesis compared to purely statistical multiplicative models [48]. Unexpectedly, our findings reveal a notable absence of these two interaction effects, which contrasts with previous studies supporting stress sensitization or inoculation models [19, 20, 23, 24]. This lack of observable interactions may be explained by the following reasons. First, the selective survival bias, wherein stress-sensitized individuals experiencing early-onset depressive symptoms or premature mortality might be excluded from our final analytical cohort, could obscure underlying interaction effects. Second, as mentioned earlier, ACEs and AAEs may represent distinct constructs in terms of the types of adversities measured, which could limit their synergistic effect on depressive symptoms. Third, China’s collectivist cultural context, characterized by its robust social support systems that reduce reliance on individual stress adaptation mechanisms, might partially mitigate the stress inoculation effects [49]. Given the heterogeneous findings across studies, our results meaningfully advance current understanding; however, conclusive inferences about the interactions between ACEs and AAEs on incident depressive symptoms cannot be substantiated.

The subgroup analyses did not detect statistically significant differences in the strength of the associations between ACEs/AAEs and incident depressive symptoms by age or sex. This suggested that, in our study population, the harmful effects of life course adversity on depressive symptoms risk were consistent across these demographic groups. In contrast, prior studies found a stronger association between ACEs and depression in females than in males [50, 51]. This discrepancy could stem from the differences in study populations or the specific types or measurements of ACEs assessed. Regarding the mediating role of AAEs, the point estimates for the indirect effect were not statistically significant in the middle-aged and female subgroups, whereas they were significant in older adults and males. However, the confidence intervals were substantially overlapped across all subgroups. This pattern indicated a lack of strong statistical evidence for meaningful heterogeneity by age or sex in this mediation pathway. Collectively, these results ​highlight critical knowledge gaps regarding age and sex effect modification, necessitating targeted mechanistic inquiry.​.

Our findings have implications for clinical practice and public health. Clinically, healthcare providers should adopt a life-course perspective by proactively screening for ACEs and AAEs in middle-age and older populations, integrating these assessments into depression risk stratification frameworks. This enables precise identification of high-risk individuals and timely delivery of preventive mental health interventions. In public health, the results delineate critical pathways for establishing a lifespan depression prevention system. Upstream strategies such as family support programs and school-based anti-bullying initiatives should be implemented to reduce the exposure to ACEs. Downstream approaches including occupational health services and community support networks can be leveraged to mitigate the occurrence of AAEs, which not only independently attenuates depression incidence but also exerts a mild effect by synergistically disrupting the ACEs-depression pathway. These evidence-informed reforms facilitate a paradigm shift from symptom management to adversity-rooted preventive strategies, offering scalable solutions to address the escalating depression burden in aging societies.

To our knowledge, this constitutes the first investigation to systematically explore the complex associations of ACEs and AAEs with incident depressive symptoms in middle-aged and older Chinese adults. The integration of prospective cohort methodology with a nationally representative sample provided two key advantages: strengthening causal inference through temporal precedence and guaranteeing statistical robustness via ample sample size.

Despite the strengths, certain constraints of the study warrant acknowledgement during results interpretation. First, ACE and AAE indicators were ascertained through retrospective self-disclosure, potentially introducing recall bias. Second, this study ​lacked multidimensional assessment​ of ACE and AAE indicators’ ​chronicity, severity, and frequency. These dimensions have been linked to adverse health outcomes in prior studies [52, 53]. Third, the present analyses aggregated ACEs or AAEs as cumulative counts, implicitly assuming effect homogeneity across adversity subtypes. This approach might obscure different risk profiles associated with specific adverse experience types. Fourth, the potential for residual confounding must be considered. Childhood socioeconomic status, a potential key confounder of the association between ACEs and later-life outcomes [54], was not sufficiently controlled for due to data limitations. Other potential confounders, such as family history of depression and personality traits, were also not taken into account [55, 56]. Fifth, some covariates adjusted for in the models (e.g., education level and chronic diseases) might lie on the causal pathway between ACEs and depressive symptoms. Treating these potential mediators as confounders could have led to an over-adjustment bias, potentially underestimating the total effect of ACEs. Sixth, follow-up disruptions and data gaps precipitated removal of significant cohort segments from the final analysis, which might lead to selection bias. As shown in Supplementary Table 2, there were significant differences in baseline characteristics between the included participants and the excluded participants. The analysis sample represented a relatively healthier and higher socioeconomic status group, which might lead to an underestimation of the strength of the association between adverse experiences and the risk of depressive symptoms. Seventh, depressive symptoms ascertainment relied on a validated screening tool rather than clinical assessments by physicians, potentially introducing misclassification bias. Lastly, the study sample was composed solely of Chinese adults, thereby constraining extrapolation of results to broader demographic contexts.​.

## Conclusions

This study demonstrated that both ACEs and AAEs were independently associated with an increased risk of incident depressive symptoms in a dose-response manner among middle-aged and older Chinese adults. AAEs partially mediated the total effect of ACEs on incident depressive symptoms. No significant multiplicative or additive interactions were observed between ACEs and AAEs on incident depressive symptoms. These findings support preventive strategies targeting ACEs and AAEs to mitigate the risk of incident depressive symptoms in later adulthood. Further studies employing rigorous causal inference approaches are needed to validate these findings.

## Supplementary Information

Supplementary Material 1

## Data Availability

The data supporting the findings of this study are available from the website of CHARLS: http://charls.pku.edu.cn. To utilize the data for research, formal approval must be secured from the Peking University CHARLS team.

## Abbreviations

ACEs: Adverse childhood experiences
AAEs: Adverse adulthood experiences
CHARLS: China Health and Retirement Longitudinal Study
KHB: Karlson-Holm-Breen
