# Supplementary material for: Complex associations of adverse childhood and adulthood experiences with incident depressive symptoms in middle-aged and older Chinese adults
Source: BMC Psychol. 2026 Mar 14;14:479. doi: 10.1186/s40359-026-04333-8 (PMC13059527; doi:10.1186/s40359-026-04333-8)
Supplement: Supplementary file 1 — Supplementary Material 1 [file 40359_2026_4333_MOESM1_ESM.docx]

**Supplementary Table 1.** Questionnaire items and responses for variables included in the ACEs and AAEs

| Domains | Questionnaire items | Answers defined as exposure to the domain | Prevalence (%) |
| --- | --- | --- | --- |
| ACEs |  |  |  |
| Physical abuse | When you were growing up, did your male/female guardian ever hit you? | Often/sometimes | 27.4 |
| Emotional neglect | How much love and affection did your female guardian give you while you were growing up? | A little/not at all | 30.3 |
|  | How much effort did your female guardian put into watching over you? | Rarely/never |  |
| Domestic violence | Have your father/mother ever beat up your mother/father? | Often/sometimes | 6.8 |
| Incarcerated household member | During the years you were growing up, have your male/female guardian ever been arrested or sent to prison? | Yes | 0.2 |
| Household substance use | During the years you were growing up, did your male/female guardian ever have alcoholism or drug? | Yes | 7.4 |
| Household mental illness | Did your male/female guardian have abnormality of mind when you were young? | Yes | 7.6 |
|  | During the years you were growing up, had your male/female guardian showed continued signs of sadness or depression that lasted two weeks or more? Was this problem of your male/female guardian, sadness or depression during all, most, some, or only a little of your childhood? | Yes, all/most |  |
| Parental separation or divorce | Were your biological parents divorced (including a long separation due to emotional problems) before you were 17 years old? | Yes | 0.3 |
| Parental disability | Did your male/female guardian have a long time be sick on bed when you were young? | Yes | 17.6 |
|  | Did your male/female guardian have a serious deformity when you were young? | Yes |  |
| Parental death^a^ | Either of the parents was dead before participant was 17 years? | Yes | 12.3 |
| Death of siblings^b^ | Any of the siblings was dead before participant was 17 years? | Yes | 13.0 |
| Unsafe neighborhood | Was it safe being out alone at night in the neighborhood where you lived as a child? | Not very safe/not safe at all | 5.8 |
| Bullying | When you were a child, how often were you picked on or bullied by kids in your neighborhood? | Often/sometimes | 13.1 |
|  | When you were a child, how often were you picked on or bullied by kids in your school? | Often/sometimes |  |
| AAEs |  |  |  |
| Death of child | Any of the child was dead? | Yes | 9.1 |
| Experienced lifetime discrimination | After you were 16 years old, because of ill health, did you experience any of the following (denied promotions, assignment to a task with fewer responsibilities, working on tasks below your qualifications, harassment by your boss or colleagues, pay cuts, dismissed)? | Yes | 7.4 |
| Ever confined to bed or home for ≥ 1 month | After you were 16 years old, because of a health condition, were you ever confined to bed or home for one month or more? | Yes | 11.7 |
| Ever hospitalized for ≥ 1 month | After you were 16 years old, because of a health condition, were you ever hospitalized for a month or more? | Yes | 9.8 |
| Ever hospitalized ≥ 3 times | Were you ever hospitalized more than three times within a 12-month period after you were 16 years old? | Yes | 2.7 |
| Ever left job for health conditions | After you were 16 years old, because of a health condition, did you leave your job for one month or more? | Yes | 14.1 |
| Physical injury | Have you ever received a physical injury that has led to any permanent handicap, disability or limitations in what you can do in daily in adulthood? | Yes | 5.8 |

Abbreviations: ACEs, adverse childhood experiences; AAEs, adverse adulthood experiences.

a Calculated based on dates of birth and their parental death.

b Calculated based on dates of birth and their sibling’s death.

**Supplementary Table 2.** Baseline characteristics of the included and excluded participants

|  | Included | Excluded | P for difference |
| --- | --- | --- | --- |
| Characteristics^a,b^ | **(n = 4516)** | **(n = 14827)** |  |
| Age, mean ± SD, years | 58.37 ± 7.97 | 59.65 ± 11.50 | < 0.001 |
| Sex, n (%) |  |  | < 0.001 |
| Male | 2440 (54.0) | 6766 (45.7) |  |
| Female | 2076 (46.0) | 8038 (54.3) |  |
| Residential area, n (%) |  |  | 0.011 |
| Urban | 1808 (40.0) | 5409 (37.9) |  |
| Rural | 2708 (60.0) | 8853 (62.1) |  |
| Marital status, n (%) |  |  | < 0.001 |
| Married | 3990 (88.4) | 11233 (78.8) |  |
| Other marital status | 526 (11.6) | 3029 (21.2) |  |
| Educational level, n (%) |  |  | < 0.001 |
| No formal education | 687 (15.2) | 4345 (29.4) |  |
| Primary school or below | 1724 (38.2) | 7273 (49.1) |  |
| Middle school | 1316 (29.1) | 2131 (14.4) |  |
| High school or above | 789 (14.5) | 1054 (7.1) |  |
| Smoking status, n (%) |  |  | < 0.001 |
| Nonsmoker | 2374 (52.6) | 8252 (58.2) |  |
| Former smoker | 768 (17.0) | 2093 (14.8) |  |
| Current smoker | 1374 (30.4) | 3833 (27.0) |  |
| Drinking status, n (%) |  |  | < 0.001 |
| Never drinker | 2250 (49.8) | 7866 (55.5) |  |
| Former drinker | 448 (9.9) | 1619 (11.4) |  |
| Current drinker | 1818 (40.3) | 4681 (33.0) |  |
| Social activity, n (%) |  |  | < 0.001 |
| No | 2095 (46.4) | 6982 (52.3) |  |
| Yes | 2421 (53.6) | 6357 (47.7) |  |
| Social support, median (IQR) | 0 (1.00) | 0 (1.00) | 0.258 |
| Chronic diseases, n (%) |  |  | < 0.001 |
| No | 1082 (24.0) | 1686 (15.6) |  |
| Yes | 3434 (76.0) | 9157 (84.5) |  |
| Retire, n (%) |  |  | < 0.001 |
| No | 3822 (84.6) | 12283 (88.4) |  |
| Yes | 694 (15.4) | 1614 (11.6) |  |
| ACEs, median (IQR) | 1.00 (2.00) | 1.00 (2.00) | < 0.001 |
| ACEs group, n (%) |  |  | < 0.001 |
| 0 | 1212 (26.8) | 1628 (22.2) |  |
| 1 | 1478 (32.7) | 2161 (29.5) |  |
| 2 | 998 (22.1) | 1635 (22.3) |  |
| 3 | 514 (11.4) | 1081 (14.7) |  |
| ≥ 4 | 314 (7.0) | 834 (11.4) |  |
| AAEs, median (IQR) | 0 (1.00) | 0 (1.00) | < 0.001 |
| AAEs group, n (%) |  |  | < 0.001 |
| 0 | 3027 (67.0) | 7515 (54.2) |  |
| 1 | 797 (17.7) | 3049 (22.0) |  |
| 2 | 327 (7.2) | 1483 (10.7) |  |
| 3 | 211 (4.7) | 1003 (7.2) |  |
| ≥ 4 | 154 (3.4) | 818 (5.9) |  |

Abbreviations: ACEs, adverse childhood experiences; AAEs, adverse adulthood experiences; SD, standard deviation; IQR, interquartile range.

a Normally distributed variables are presented as mean ± SD, non-normally distributed variables are presented as median (IQR), and categorical variables are presented as n (%).

b Missing data in the group of excluded participants: 592 for age, 23 for sex, 565 for residential area, 565 for marital status, 24 for educational level, 649 for smoking status, 661 for drinking status, 1488 for social activity, 888 for social support, 3984 for chronic diseases, 930 for retire, 7488 for ACEs and ACEs group, 959 for AAEs and AAEs group.

**Supplementary Table 3.** Baseline characteristics of the study population by the number of ACEs

|  | Overall | Number of ACEs indicators | | | | | P for difference | P for trend |
| --- | --- | --- | --- | --- | --- | --- | --- | --- |
| Characteristics^a^ | **(n = 4516)** | **0 (n = 1212)** | **1 (n = 1478)** | **2 (n = 998)** | **3 (n = 514)** | **≥ 4 (n = 314)** |  |  |
| Age, mean ± SD, years | 58.37 ± 7.97 | 57.83 ± 7.68 | 58.40 ± 8.07 | 58.41 ± 7.91 | 58.99 ± 8.36 | 59.13 ± 8.05 | 0.020 | 0.003 |
| Sex, n (%) |  |  |  |  |  |  | < 0.001 | < 0.001 |
| Male | 2440 (54.0) | 585 (48.3) | 803 (54.3) | 552 (55.3) | 326 (63.4) | 174 (55.4) |  |  |
| Female | 2076 (46.0) | 627 (51.7) | 675 (45.7) | 446 (44.7) | 188 (36.6) | 140 (44.6) |  |  |
| Residential area, n (%) |  |  |  |  |  |  | 0.205 | 0.610 |
| Urban | 1808 (40.0) | 478 (39.4) | 606 (41.0) | 396 (39.7) | 219 (42.6) | 109 (34.7) |  |  |
| Rural | 2708 (60.0) | 734 (60.6) | 872 (59.0) | 602 (60.3) | 295 (57.4) | 205 (65.3) |  |  |
| Marital status, n (%) |  |  |  |  |  |  | 0.083 | 0.010 |
| Married | 3990 (88.4) | 1090 (89.9) | 1313 (88.8) | 867 (86.9) | 452 (87.9) | 268 (85.4) |  |  |
| Other marital status | 526 (11.6) | 122 (10.1) | 165 (11.2) | 131 (13.1) | 62 (12.1) | 46 (14.6) |  |  |
| Educational level, n (%) |  |  |  |  |  |  | 0.029 | < 0.001 |
| No formal education | 687 (15.2) | 171 (14.1) | 223 (15.1) | 158 (15.8) | 82 (16.0) | 53 (16.9) |  |  |
| Primary school or below | 1724 (38.2) | 424 (35.0) | 556 (37.6) | 394 (39.5) | 211 (41.1) | 139 (44.3) |  |  |
| Middle school | 1316 (29.1) | 386 (31.8) | 431 (29.2) | 283 (28.4) | 134 (26.1) | 82 (26.1) |  |  |
| High school or above | 789 (14.5) | 231 (19.1) | 268 (18.1) | 163 (16.3) | 87 (16.9) | 40 (12.7) |  |  |
| Smoking status, n (%) |  |  |  |  |  |  | < 0.001 | < 0.001 |
| Nonsmoker | 2374 (52.6) | 706 (58.3) | 771 (52.2) | 503 (50.4) | 233 (45.3) | 161 (51.3) |  |  |
| Former smoker | 768 (17.0) | 178 (14.7) | 263 (17.8) | 164 (16.4) | 108 (21.0) | 55 (17.5) |  |  |
| Current smoker | 1374 (30.4) | 328 (27.1) | 444 (30.0) | 331 (33.2) | 173 (33.7) | 98 (31.2) |  |  |
| Drinking status, n (%) |  |  |  |  |  |  | < 0.001 | < 0.001 |
| Never drinker | 2250 (49.8) | 671 (55.4) | 752 (50.9) | 473 (47.4) | 214 (41.6) | 140 (44.6) |  |  |
| Former drinker | 448 (9.9) | 105 (8.7) | 149 (10.1) | 101 (10.1) | 56 (10.9) | 37 (11.8) |  |  |
| Current drinker | 1818 (40.3) | 436 (36.0) | 577 (39.0) | 424 (42.5) | 244 (47.5) | 137 (43.6) |  |  |
| Social activity, n (%) |  |  |  |  |  |  | 0.468 | 0.834 |
| No | 2095 (46.4) | 567 (46.8) | 662 (44.8) | 484 (48.5) | 240 (46.7) | 142 (45.2) |  |  |
| Yes | 2421 (53.6) | 645 (53.2) | 816 (55.2) | 514 (51.5) | 274 (53.3) | 172 (54.8) |  |  |
| Social support, median (IQR) | 0 (1.00) | 0 (0) | 0 (1.00) | 0 (1.00) | 0 (1.00) | 0 (1.00) | < 0.001 | < 0.001 |
| Chronic diseases, n (%) |  |  |  |  |  |  | < 0.001 | < 0.001 |
| No | 1082 (24.0) | 333 (27.5) | 379 (25.6) | 207 (20.7) | 107 (20.8) | 56 (17.8) |  |  |
| Yes | 3434 (76.0) | 879 (72.5) | 1099 (74.4) | 791 (79.3) | 407 (79.2) | 258 (82.2) |  |  |
| Retire, n (%) |  |  |  |  |  |  | 0.383 | 0.831 |
| No | 3822 (84.6) | 1035 (85.4) | 1234 (83.5) | 858 (86.0) | 428 (83.3) | 267 (85.0) |  |  |
| Yes | 694 (15.4) | 177 (14.6) | 244 (16.5) | 140 (14.0) | 86 (16.7) | 47 (15.0) |  |  |

Abbreviations: ACEs, adverse childhood experiences; SD, standard deviation; IQR, interquartile range.

a Normally distributed variables are presented as mean ± SD, non-normally distributed variables are presented as median (IQR), and categorical variables are presented as n (%).

**Supplementary Table 4.** Baseline characteristics of the study population by the number of AAEs

|  | Overall | Number of AAEs indicators | | | | | P for difference | P for trend |
| --- | --- | --- | --- | --- | --- | --- | --- | --- |
| Characteristics^a^ | **(n = 4516)** | **0 (n = 3027)** | **1 (n = 797)** | **2 (n = 327)** | **3 (n = 211)** | **≥ 4 (n = 154)** |  |  |
| Age, mean ± SD, years | 58.37 ± 7.97 | 57.63 ± 7.66 | 59.98 ± 8.61 | 59.94 ± 8.15 | 59.12 ± 8.02 | 60.01 ± 8.05 | < 0.001 | 0.007 |
| Sex, n (%) |  |  |  |  |  |  | < 0.001 | < 0.001 |
| Male | 2440 (54.0) | 1552 (51.3) | 456 (57.2) | 193 (59.0) | 126 (59.7) | 113 (73.4) |  |  |
| Female | 2076 (46.0) | 1475 (48.7) | 341 (42.8) | 134 (41.0) | 85 (40.3) | 41 (26.6) |  |  |
| Residential area, n (%) |  |  |  |  |  |  | 0.027 | 0.011 |
| Urban | 1808 (40.0) | 1262 (41.7) | 288 (36.1) | 125 (38.2) | 76 (36.0) | 57 (37.0) |  |  |
| Rural | 2708 (60.0) | 1765 (58.3) | 509 (63.9) | 292 (61,8) | 135 (64.0) | 97 (63.0) |  |  |
| Marital status, n (%) |  |  |  |  |  |  | 0.041 | 0.006 |
| Married | 3990 (88.4) | 2706 (89.4) | 688 (86.3) | 283 (86.5) | 182 (86.3) | 131 (85.1) |  |  |
| Other marital status | 526 (11.6) | 321 (10.6) | 109 (13.7) | 44 (13.5) | 29 (13.7) | 23 (14.9) |  |  |
| Educational level, n (%) |  |  |  |  |  |  | < 0.001 | < 0.001 |
| No formal education | 687 (15.2) | 421 (13.9) | 144 (18.1) | 61 (18.7) | 34 (16.1) | 27 (17.5) |  |  |
| Primary school or below | 1724 (38.2) | 1102 (36.4) | 330 (41.4) | 133 (40.7) | 92 (43.6) | 67 (43.5) |  |  |
| Middle school | 1316 (29.1) | 937 (31.0) | 213 (26.7) | 80 (24.5) | 47 (22.3) | 39 (25.3) |  |  |
| High school or above | 789 (14.5) | 567 (18.7) | 110 (13.8) | 53 (16.2) | 38 (18.0) | 21 (13.6) |  |  |
| Smoking status, n (%) |  |  |  |  |  |  | < 0.001 | < 0.001 |
| Nonsmoker | 2374 (52.6) | 1675 (55.3) | 398 (49.9) | 150 (45.9) | 95 (45.0) | 56 (36.4) |  |  |
| Former smoker | 768 (17.0) | 470 (15.5) | 139 (17.4) | 69 (21.1) | 46 (21.8) | 44 (28.6) |  |  |
| Current smoker | 1374 (30.4) | 882 (29.1) | 260 (32.6) | 198 (33.0) | 70 (33.2) | 54 (35.1) |  |  |
| Drinking status, n (%) |  |  |  |  |  |  | < 0.001 | < 0.001 |
| Never drinker | 2250 (49.8) | 1593 (52.6) | 378 (47.4) | 135 (41.3) | 88 (41.7) | 56 (36.4) |  |  |
| Former drinker | 448 (9.9) | 245 (8.1) | 89 (11.2) | 50 (15.3) | 35 (16.6) | 29 (18.8) |  |  |
| Current drinker | 1818 (40.3) | 1189 (39.3) | 330 (41.4) | 142 (43.4) | 88 (41.7) | 69 (44.8) |  |  |
| Social activity, n (%) |  |  |  |  |  |  | 0.367 | 0.808 |
| No | 2095 (46.4) | 1401 (46.3) | 374 (46.9) | 153 (46.8) | 87 (41.2) | 80 (51.9) |  |  |
| Yes | 2421 (53.6) | 1626 (53.7) | 423 (53.1) | 174 (53.2) | 124 (58.8) | 74 (48.1) |  |  |
| Social support, median (IQR) | 0 (1.00) | 0 (1.00) | 0 (1.00) | 0 (1.00) | 0 (1.00) | 0 (1.00) | 0.020 | 0.025 |
| Chronic diseases, n (%) |  |  |  |  |  |  | < 0.001 | < 0.001 |
| No | 1082 (24.0) | 823 (27.2) | 160 (20.1) | 52 (15.9) | 31 (14.7) | 16 (10.4) |  |  |
| Yes | 3434 (76.0) | 2204 (72.8) | 637 (79.9) | 275 (84.1) | 180 (85.3) | 138 (89.6) |  |  |
| Retire, n (%) |  |  |  |  |  |  | 0.324 | 0.216 |
| No | 3822 (84.6) | 2570 (84.9) | 683 (85.7) | 265 (81.0) | 175 (82.9) | 129 (83.8) |  |  |
| Yes | 694 (15.4) | 457 (15.1) | 114 (14.3) | 62 (19.0) | 36 (17.1) | 25 (16.2) |  |  |

Abbreviations: AAEs, adverse adulthood experiences; SD, standard deviation; IQR, interquartile range.

a Normally distributed variables are presented as mean ± SD, non-normally distributed variables are presented as median (IQR), and categorical variables are presented as n (%).

**Supplementary Table 5.** Mediating role of AAEs in the association between high-dose ACEs (≥ 4) and incident depressive symptoms

|  | RR (95% CI)^a^ | | | Mediation proportion, % |
| --- | --- | --- | --- | --- |
|  | **Total effect** | **Direct effect** | **Indirect effect** |  |
| No. of ACEs indicators |  |  |  |  |
| 0 | 1.00 (reference) | 1.00 (reference) | 1.00 (reference) |  |
| ≥ 4 | 1.70 (1.44, 2.02) | 1.65 (1.34, 2.05) | 1.03 (1.003, 1.05) | 7.02 |

Abbreviations: AAEs, adverse adulthood experiences; ACEs, adverse childhood experiences; RR, relative risk; 95% CI, 95% confidence interval.

a Models were adjusted for age, sex, residential area, marital status, education level, smoking status, drinking status, social activity participation, presence of chronic diseases, retirement status, and adulthood social support.

**Supplementary Table 6.** Associations of ACEs and AAEs with incident depressive symptoms: subgroup analysis

|  | RR (95% CI)^a^ | | | |
| --- | --- | --- | --- | --- |
|  | **Aged < 60 years** | **Aged ≥ 60 years** | **Male** | **Female** |
| ACEs indicators^b^ (1-unit per increasing) | 1.14 (1.09, 1.19) | 1.09 (1.03, 1.15) | 1.16 (1.09, 1.22) | 1.10 (1.05, 1.15) |
| P for interaction | 0.201 | | 0.953 | |
| No. of ACEs indicators |  |  |  |  |
| 0 | 1.00 (reference) | 1.00 (reference) | 1.00 (reference) | 1.00 (reference) |
| 1 | 1.11 (0.92, 1.34) | 0.95 (0.78, 1.15) | 1.02 (0.80, 1.30) | 1.05 (0.90, 1.24) |
| 2 | 1.37 (1.13, 1.66) | 1.04 (0.85, 1.28) | 1.34 (1.05, 1.71) | 1.15 (0.97, 1.37) |
| 3 | 1.34 (1.06, 1.70) | 1.27 (1.00, 1.61) | 1.46 (1.12, 1.90) | 1.22 (0.98, 1.52) |
| ≥ 4 | 2.02 (1.60, 2.53) | 1.39 (1.09, 1.78) | 1.84 (1.38, 2.46) | 1.64 (1.34, 2.01) |
| P for trend | < 0.001 | 0.002 | < 0.001 | < 0.001 |
| P for interaction | 0.118 | | 0.915 | |
| AAEs indicators^b^ (1-unit per increasing) | 1.10 (1.04, 1.16) | 1.10 (1.04, 1.16) | 1.12 (1.05, 1.19) | 1.08 (1.03, 1.14) |
| P for interaction | 0.111 | | 0.243 | |
| No. of AAEs indicators |  |  |  |  |
| 0 | 1.00 (reference) | 1.00 (reference) | 1.00 (reference) | 1.00 (reference) |
| 1 | 1.16 (0.97, 1.39) | 1.10 (0.92, 1.31) | 1.10 (0.89, 1.36) | 1.16 (1.00, 1.36) |
| 2 | 1.34 (1.05, 1.71) | 0.98 (0.75, 1.28) | 1.35 (1.03, 1.77) | 1.01 (0.79, 1.29) |
| 3 | 1.14 (0.83, 1.57) | 1.32 (1.07, 1.73) | 1.29 (0.95, 1.76) | 1.21 (0.92, 1.59) |
| ≥ 4 | 1.49 (1.09, 2.04) | 1.62 (1.23, 2.14) | 1.57 (1.16, 2.11) | 1.61 (1.23, 2.09) |
| P for trend | 0.002 | 0.002 | < 0.001 | 0.004 |
| P for interaction | 0.123 | | 0.273 | |

Abbreviations: ACEs, adverse childhood experiences; AAEs, adverse adulthood experiences; RR, relative risk; 95% CI, 95% confidence interval.

a Models were adjusted for age, sex, residential area, marital status, education level, smoking status, drinking status, social activity participation, presence of chronic diseases, retirement status, and adulthood social support.

**Supplementary Table 7.** Mediating role of AAEs in the association between ACEs and incident depressive symptoms: subgroup analysis

|  | RR (95% CI)^a^ | | | Mediation proportion (%) |
| --- | --- | --- | --- | --- |
|  | **Total effect** | **Direct effect** | **Indirect effect** |  |
| Aged < 60 years |  |  |  |  |
| ACEs indicators^b^ (1-unit per increasing) | 1.14 (1.08, 1.21) | 1.13 (1.07, 1.20) | 1.01 (1.00, 1.01) | NA |
| No. of ACEs indicators |  |  |  |  |
| 0 | 1.00 (reference) | 1.00 (reference) | 1.00 (reference) |  |
| ≥ 1 | 1.29 (1.07, 1.56) | 1.28 (1.06, 1.54) | 1.01 (1.00, 1.02) | NA |
| Aged ≥ 60 years |  |  |  |  |
| ACEs indicators^b^ (1-unit per increasing) | 1.09 (1.02, 1.16) | 1.08 (1.01, 1.15) | 1.01 (1.001, 1.01) | 7.66 |
| No. of ACEs indicators |  |  |  |  |
| 0 | 1.00 (reference) | 1.00 (reference) | 1.00 (reference) |  |
| ≥ 1 | NA^c^ | NA^c^ | NA^c^ | NA^c^ |
| Male |  |  |  |  |
| ACEs indicators^b^ (1-unit per increasing) | 1.15 (1.08, 1.23) | 1.15 (1.07, 1.23) | 1.01 (1.001, 1.01) | 4.85 |
| No. of ACEs indicators |  |  |  |  |
| 0 | 1.00 (reference) | 1.00 (reference) | 1.00 (reference) |  |
| ≥ 1 | 1.27 (1.01, 1.60) | 1.25 (0.99, 1.57) | 1.02 (1.00, 1.03) | NA |
| Female |  |  |  |  |
| ACEs indicators^b^ (1-unit per increasing) | 1.10 (1.04, 1.16) | 1.09 (1.03, 1.15) | 1.01 (1.00, 1.01) | NA |
| No. of ACEs indicators |  |  |  |  |
| 0 | 1.00 (reference) | 1.00 (reference) | 1.00 (reference) |  |
| ≥ 1 | NA^c^ | NA^c^ | NA^c^ | NA^c^ |

Abbreviations: AAEs, adverse adulthood experiences; ACEs, adverse childhood experiences; RR, relative risk; 95% CI, 95% confidence interval; NA, not available or not applicable.

a Models were adjusted for age, sex, residential area, marital status, education level, smoking status, drinking status, social activity participation, presence of chronic diseases, retirement status, and adulthood social support.

b Continuous variable.

c Mediation analysis was not conducted since the total association was not significant.

**Supplementary Table 8.** Interaction analysis of ACEs and AAEs on incident depressive symptoms: subgroup analysis

|  | RR (95% CI)^a^ | | | |
| --- | --- | --- | --- | --- |
| Interaction | **Aged < 60 years** | **Aged ≥ 60 years** | **Male** | **Female** |
| Multiplicative interaction |  |  |  |  |
| Product term (ACEs × AAEs) | 1.09 (0.76, 1.55) | 0.94 (0.67, 1.31) | 1.01 (0.66, 1.55) | 0.91 (0.68, 1.22) |
| Additive interaction |  |  |  |  |
| RERI | 0.15 (-0.25, 0.56) | -0.07 (-0.45, 0.32) | 0.07 (-0.42, 0.57) | -0.09 (-0.45, 0.27) |
| AP | 0.10 (-0.16, 0.37) | -0.05 (-0.37, 0.26) | 0.05 (-0.28, 0.38) | -0.07 (-0.34, 0.20) |
| SI | 1.41 (0.47, 4.22) | 0.77 (0.21, 2.79) | 1.16 (0.37, 3.60) | 0.78 (0.34, 1.83) |

Abbreviations: ACEs, adverse childhood experiences; AAEs, adverse adulthood experiences; RR, relative risk; 95% CI, 95% confidence interval, RERI, relative excess risk due to interaction; AP, attributable proportion; SI, synergy index.

a Models were adjusted for age, sex, residential area, marital status, education level, smoking status, drinking status, social activity participation, presence of chronic diseases, retirement status, and adulthood social support.
